# Consequences of life history switch point plasticity for juvenile morphology and locomotion in the *Túngara frog*

**DOI:** 10.7717/peerj.1268

**Published:** 2015-09-22

**Authors:** Julie F. Charbonnier, James R. Vonesh

**Affiliations:** Department of Biology, Virginia Commonwealth University, Richmond, VA, USA

**Keywords:** Carry-over effects, Hydroperiod, Locomotion, Metamorphosis, Plasticity

## Abstract

Many animals with complex life cycles can cope with environmental uncertainty by altering the timing of life history switch points through plasticity. Pond hydroperiod has important consequences for the fitness of aquatic organisms and many taxa alter the timing of life history switch points in response to habitat desiccation. For example, larval amphibians can metamorphose early to escape drying ponds. Such plasticity may induce variation in size and morphology of juveniles which can result in carry-over effects on jumping performance. To investigate the carry-over effects of metamorphic plasticity to pond drying, we studied the Túngara frog, *Physalaemus pustulosus*, a tropical anuran that breeds in highly ephemeral habitats. We conducted an outdoor field mesocosm experiment in which we manipulated water depth and desiccation and measured time and size at metamorphosis, tibiofibula length and jumping performance. We also conducted a complimentary laboratory experiment in which we manipulated resources, water depth and desiccation. In the field experiment, metamorphs from dry-down treatments emerged earlier, but at a similar size to metamorphs from constant depth treatments. In the laboratory experiment, metamorphs from the low depth and dry-down treatments emerged earlier and smaller. In both experiments, frogs from dry-down treatments had relatively shorter legs, which negatively impacted their absolute jumping performance. In contrast, reductions in resources delayed and reduced size at metamorphosis, but had no negative effect on jumping performance. To place these results in a broader context, we review past studies on carry-over effects of the larval environment on jumping performance. Reductions in mass and limb length generally resulted in lower jumping performance across juvenile anurans tested to date. Understanding the consequences of plasticity on size, morphology and performance can elucidate the linkages between life stages.

## Introduction

Many organisms have complex life cycles and pass through ecologically distinct phases during ontogeny (Moran, 1994). During ontogenetic switch points, organisms undergo dramatic changes in physiology, morphology, behavior, and relocate to new habitats (Wilbur, 1980; Moran, 1994). Organisms can alter the timing of life history switch points through developmental plasticity, enabling them to respond to environmental heterogeneity while optimizing survival and size across life stages (Werner, 1986). Complex life history strategies should theoretically facilitate modularity and independence between life stages and environments (Ebenman, 1992; Moran, 1994). However, a large body of literature shows that distinct life stages are highly linked (Mousseau & Fox, 1998; Podolsky & Moran, 2006; Pechenik, 2006; Marshall & Morgan, 2011). Accelerating or delaying life history switch points requires modifying growth and development processes, constraining the phenotype in the next life stage (Smith-Gill & Berven, 1979; Gomez-Mestre et al., 2010). These phenotypic effects are defined as carry-over effects, trait differences which originate in one life stage and persist or are expressed in subsequent stages (Pechenik, 2006). Early environmental variation (e.g., resource availability, predation risk) may have similar impacts on the life history switch point but result in different carry-over effects, depending on how they modulate relative growth versus developmental pathways (Blouin & Loeb, 1991; Leips & Travis, 1994; Gomez-Mestre et al., 2010).

The interplay between growth and development in early life stages is particularly important for understanding carry-over effects in amphibians. Larval amphibians are sensitive to environmental variation, and their growth and developmental rates can be largely decoupled and altered in response to different risks during ontogeny (Smith-Gill & Gill, 1978; Smith-Gill & Berven, 1979; Leips & Travis, 1994; Benard, 2004; Richter-Boix, Tejedo & Rezende, 2011). For example, both pond drying and resource limitation have been shown to have differing impacts on growth and development (Travis, 1984; Blouin & Loeb, 1991; Leips & Travis, 1994; Tejedo & Reques, 1994; Michimae & Emura, 2012). Anuran larvae accelerate metamorphosis to escape drying ponds (Newman, 1989; Newman, 1992; Denver, Mirhadi & Phillips, 1998; Richter-Boix, Llorente & Montori, 2006; Richter-Boix, Tejedo & Rezende, 2011) by stimulating hormonal pathways (Denver, 1997; Denver, 2013; Denver, Mirhadi & Phillips, 1998; Gomez-Mestre, Kulkarni & Buchholz, 2013). Accelerated metamorphosis can result in reduced body size (Laurila & Kujasalo, 1999; Richter-Boix, Llorente & Albert, 2006; Richter-Boix, Tejedo & Rezende, 2011) and smaller hindlimb length (Richter-Boix, Llorente & Albert, 2006; Johansson, Lederer & Lind, 2010; Tejedo et al., 2010), and can influence other physiological traits (immune function; Gervasi & Foufopoulos, 2007; oxidative stress; Gomez-Mestre, Kulkarni & Buchholz, 2013). Food limitation or high conspecific density results in smaller size at metamorphosis (John-Alder & Morin, 1990; Goater, Semlitsch & Bernasconi, 1993; Newman, 1994; Tejedo, Semlitsch & Hotz, 2000a) and relatively shorter legs (Relyea & Hoverman, 2003; Gomez-Mestre et al., 2010; Tejedo et al., 2010). When pond drying and resource limitation occur simultaneously, this can result in diverse and complementary or contradictory carry-over effects on size and morphology at metamorphosis (Tejedo & Reques, 1994; Michimae & Emura, 2012; Enriquez-Urzelai et al., 2013). For example, larvae in resource limited, ephemeral environments may simultaneously experience reduction in hindlimb length due to resource limitation and pond-drying, while larvae in ephemeral, resource rich environments may be able to mitigate the carry-over effects due to their high growth rate.

Changes in metamorphic phenotype arising from variation in the larval environment can limit ecologically relevant performance capacities of juveniles such as locomotion (Arnold, 1983; Irschick & Garland, 2001). In amphibians, jumping performance influences food acquisition, predator avoidance, and dispersal capacities (Wassersug & Sperry, 1977; Walton, 1988; Heinen & Hammond, 1997; Phillips et al., 2006; Patrick et al., 2008; Ward-Fear, Brown & Shine, 2010). In adult frogs, both larger size and longer relative hindlimbs should enhance jumping performance (Rand, 1952; Zug, 1972; Emerson, 1978). If the juvenile morphology-performance relationship is similar to what has been observed in adults, we would predict that small juveniles with short relative leg length would have the poorest performance (Zug, 1972; Emerson, 1978). Carry-over effects on size and morphology may act synergistically to influence performance capacities. Alternatively, juveniles may possess compensatory mechanisms to buffer the effects of past developmental histories on performance (Carrier, 1996) or changes in morphology may be too small to result in changes in performance (Emerson, Travis & Blouin, 1988; Gomez-Mestre et al., 2010; Tejedo et al., 2010). The relationships between life history plasticity, carry-over effects on morphology and size, and subsequent juvenile jumping performance are difficult to predict given the complexity created by competing effects. To address this complexity, we quantitatively summarize past experimental studies that have manipulated characteristics of the larval environment to explore the relationship between body size, hindlimb length and metamorphic jumping performance in juvenile anurans.

The objective of this study is to test how plastic responses to environmental variation in an early life stage influence size and morphology in subsequent stages, and how these effects interact to mediate juvenile locomotion. Since the effects of life history plasticity may be dependent on how growth and development rates (and their interaction) are modulated, we manipulated environmental factors expected to impact growth (resources levels) and development (pond drying). We used the Túngara frog (*Physalaemus pustulosus*), a Neotropical species that inhabits ephemeral and shallow habitats that may dry completely during the rainy season (Marsh, Fegraus & Harrison, 1999). We conducted complementary outdoor mesocosm and laboratory experiments to delineate how pond drying and resource availability influence juvenile time to metamorphosis, size, hindlimb length, and jumping performance. Since larval survival and growth trajectories are important for interpreting patterns at metamorphosis, we also measured growth and survival during the larval stage. We predicted that pond drying would result in accelerated metamorphosis which would have negative effects on size, hindlimb length, and jumping performance. We hypothesized that these negative effects would be mitigated by high resource levels and exacerbated in low resource conditions.

## Methods

### Study species and animal collection

This study was conducted at the Smithsonian Tropical Research Institute in Gamboa, Panama (9°17′17″N, 79°42′11″W) between 17-June and 4-Aug-2011. Our study species, the Túngara frogs (*Engystomops* = *Physalaemus pustulosus*, Family: Leptodactylidae) is found throughout Central America and are locally abundant at this study site. Eggs are laid in foam nests (clutch size: 234 eggs ± 97.6, mean ± SE, (Ryan, 1985)) and hatch within 2–4 days. Five foam nests were collected immediately after laying on 17-June-2011 for both field and laboratory experiments. Egg nests were kept in separate containers until hatching in an ambient-temperature laboratory. This research was conducted under STRI’s IACUC protocol 2011-0616-2014-04.

### Field mesocosm experiment

We conducted an outdoor mesocosm experiment to determine how pond drying may impact development and post-metamorphic performance, given ambient variation in daily temperature and water levels. To distinguish between the effects of water depth versus desiccation, we included two control treatments: a constant high depth treatment (10 cm of water, 15.2 L) and a constant low depth treatment (2 cm, 3 L). Each treatment received fifteen tadpoles (3 days post hatching). Our dry-down treatment started at 10 cm (15.2 L) and finished at 1 cm depth (1.5 L). Water levels were reduced by 1 cm d^−2^ for 19 days and subsequently maintained at 1 cm until all surviving metamorphs had emerged (20 d). Water was disturbed in control treatments to mimic disturbance associated with water reduction. Treatments were replicated ten times and randomly assigned to 60 L plastic tub containers (40 cm deep × 44 cm diameter) arranged in three rows of ten tubs in a partially forested canopy field. Tubs were filled with a mix of filtered aged rain and tap water and stocked with 10 *Inga sp.* tree leaves for cover and with 50 ml of concentrated marsh inoculate. Marsh inoculate was collected by repeatedly sweeping a plankton net through the water column of Kent’s Marsh (9°07′13″N, 79°41′46″W) and then filtering it through a 1 mm mesh filter to exclude macroinvertebrates. Tanks were covered with fine nylon mesh and secured with elastic bands to prevent colonization by other organisms. Resources were supplemented with 0.27 g commercial rabbit food (primarily alfalfa, ∼17% protein) weekly for the first two weeks and twice weekly thereafter. All tadpoles were dorsally photographed and digitally measured using ImageJ software (Schneider, Rasband & Eliceiri, 2012) at the start of experiment to test that there were no differences in initial length among treatments and two weeks after the start of the experiment to assess larval growth. Tanks were checked daily and maintained at experimental water depth. Metamorphs were removed upon forelimb emergence (stage 42 (Gosner, 1960)). Since water temperatures may influence amphibian development (Newman, 1989; Gomez-Mestre et al., 2010), we randomly assigned three HOBO data loggers per treatment (*n* = 9) to monitor temperature at half hour intervals.

### Laboratory experiment

To determine how resource availability and pond drying jointly impact post-metamorphic performance, we conducted a 2 × 3 factorial experiment where we manipulated resource levels (high, low food) by water depth (high, low, dry-down). Lab treatments were conducted in 1.5 L (15.7 cm × 10.9 cm) plastic containers filled with aged tap water and replicated eight times. Each container received 1 tadpole (4 d post-hatching). Treatment depths paralleled the field experiment: constant high depth (10 cm of water, 1.5 L), constant low depth (2 cm, 0.35 L) and a dry-down treatment (10 to 1 cm over 19 d, 1.5–0.17 L). Tadpoles in high resource treatments were initially fed 0.02 g d^−1^ *Nutrafin Max*^®^ fish food (∼44% protein) for the first week then 0.08 g d^−1^ until end of experiment. Tadpoles in low resource treatments were fed the same amount but every two days. Although food types differed in quality between experiments, *Nutrafin Max*^®^ flakes enabled us to allot smaller quantities of food per tadpole, and per capita rates in the laboratory low resource treatments were similar to per capita rates in the field treatment (Field: 0.36 g wk^−1^ indiv^−1^, High resources laboratory: 0.60 g wk^−1^ indiv^−1^, Low resources laboratory: 0.30 g wk^−1^ indiv^−1^). Feces and unconsumed food were removed every other day and water was changed weekly. Laboratory conditions were 12:12 photoperiod cycle in an ambient temperature laboratory.

### Post-metamorphic jumping performance

Metamorphs were collected daily at forelimb emergence (stage 42, (Gosner, 1960)) and weighed (±0.001 g). Metamorphs were maintained individually in 240 ml plastic cups in the laboratory until complete tail resorption (stage 46, (Gosner, 1960)). Upon tail resorption (2–3 days), we measured snout-vent length (SVL) and tibiofibula length using digital calipers and reweighed frogs. To assess jumping performance, each frog was stimulated to jump in a circular array with 0.5 cm markings. Jumping was initiated by lightly prodding the frog’s urostyle and estimated visually (Goater, Semlitsch & Bernasconi, 1993; Niehaus, Wilson & Franklin, 2006). Frogs were jumped three times per trial and three trials were conducted per frog (for a total of nine jumps), with a 5 min rest period in between trials. We estimated repeatability of locomotion using the intraclass correlation (Watkins, 2001). A subset of 24 frogs were jumped 21 times to examine whether nine jumps accurately estimated maximum and average jumping performance as measured based on a larger sample (maximum jump: *F*_1,22_ = 337.6, *P* < 0.001, *R*^2^ = 0.94, *n* = 24, average: *F*_1,22_ = 249.2, *P* < 0.001, *R*^2^ = 0.92, *n* = 24).

### Statistical analyses

All analyses were conducted on tank means using R version 2.14.0 (R Core Team, 2015). Maximum jump length did not meet assumptions of normality and was log-transformed. Larval growth was measured as the difference between the initial measurement of tadpoles on day 1 of the experiment and tadpole length after 14 days in the aquatic tanks. We analyzed larval growth using an analysis of variance (ANOVA). We analyzed percent survival using a generalized linear model with a binomial distribution. To address effects of treatments on metamorphic phenotype, we conducted a multivariate analysis of variance (MANOVA) including the response variables time to metamorphosis, SVL, tibiofibula length and final mass at metamorphosis. Post-hoc comparisons were conducted using Tukey’s HSD. We compared tibiofibula length relative to SVL using two-factors analysis of covariance (ANCOVA) with experimental treatments as factors and SVL as a covariate. To analyze locomotor performance, relative leg length was calculated by taking the residual scores from a linear regression of tibiofibula length against SVL (Phillips et al., 2006; Llewelyn et al., 2010). We then analyzed maximum jump length with treatments as factor and SVL and relative leg length as covariates.

### Meta-analysis

We surveyed the literature using search engines (Web of Science and Google Scholar) for published studies in English that manipulated the larval environment and quantified effects on metamorph size, morphology and jumping performance in amphibians from 1900–2013. We performed searches using the keywords: “morphology”, “tibiofibula”, “jump*”, “locomot*” and cross referenced available studies. In order to be included, studies had to manipulate the larval environment in a laboratory or outdoor mesocosm experiment and report effects on at least juvenile size (mass) and maximum jump length after metamorphosis (see Supplemental Information 2). Data were extracted from the text and tables or measured from figures using ImageJ software. We used the natural log of the response ratio (LRR) to calculate proportional change in mean mass, jump length and tibiofibula length between control and experimental treatments. The response ratio is a common metric used in meta-analysis which quantifies the proportionate change generated by the experimental manipulation (Hedges, Gurevitch & Curtis, 1999). Experimental treatments were never used more than once as this would increase non-independence among the effect (Gurevitch et al., 1992; Skelly, 2002). We conducted stepwise linear regression on log response ratio of jump length with LRR of mass and tibiofibula as predictor variables to quantify how a proportional change in mass and tibiofibula length resulted in a proportional change in jump length.

## Results

### Effects on larval growth and survival

#### Field experiment

Mean daily water temperature from 23 June–16 July was 26.7 °C ± 0.12 did not differ between treatments (*F*_2,6_ = 1.5, *P* = 0.29). Maximum daily temperature differed between treatments (*F*_2,6_ = 5.2, *P* = 0.049) but only between dry-down (33.4 °C ± 1.5) and high depth (30. 9 °C ± 0.62) treatments (*P* = 0.04). Tadpole length did not differ among treatments at the start of the experiment (*F*_2,25_ = 0.24, *P* = 0.79). Tadpoles in low depth treatments had on average 12% lower growth during the first week of the experiment than high depth and dry-down treatments (*F*_2,23_ = 4.59, *P* = 0.02; Supplemental Information). Mean survivorship through metamorphosis was high (90 ± 10%), although low depth treatments had 5% higher survival than high depth and dry-down treatments (*χ*^2^ = 6.3, *P* = 0.04). One replicate of each of the constant depth treatments was lost due to experimental error (*n* = 10 for dry-down treatments, *n* = 9 for high and low depth treatments).

#### Laboratory experiment

Mean daily temperature in the laboratory was 26.4 °C ± 0. 26. Initial length of tadpoles did not differ between treatments (*F*_5,42_ = 1.68, *P* = 0.16). Only resources affected growth during the first week of the experiment (*F*_1,43_ = 36.4, *P* < 0.001), with frogs in high resource treatments growing 36% faster than frogs from low resource treatments (Supplemental Information). There was no difference in survival among treatments (*χ*^2^ = 4.2, *P* = 0.52). All frogs survived to forelimb emergence (Gosner Stage 42) and 83% survived until tail reabsorption (Gosner Stage 46).

### Effects on metamorphic phenotype and jumping performance

#### Field experiment

**Figure 1:**
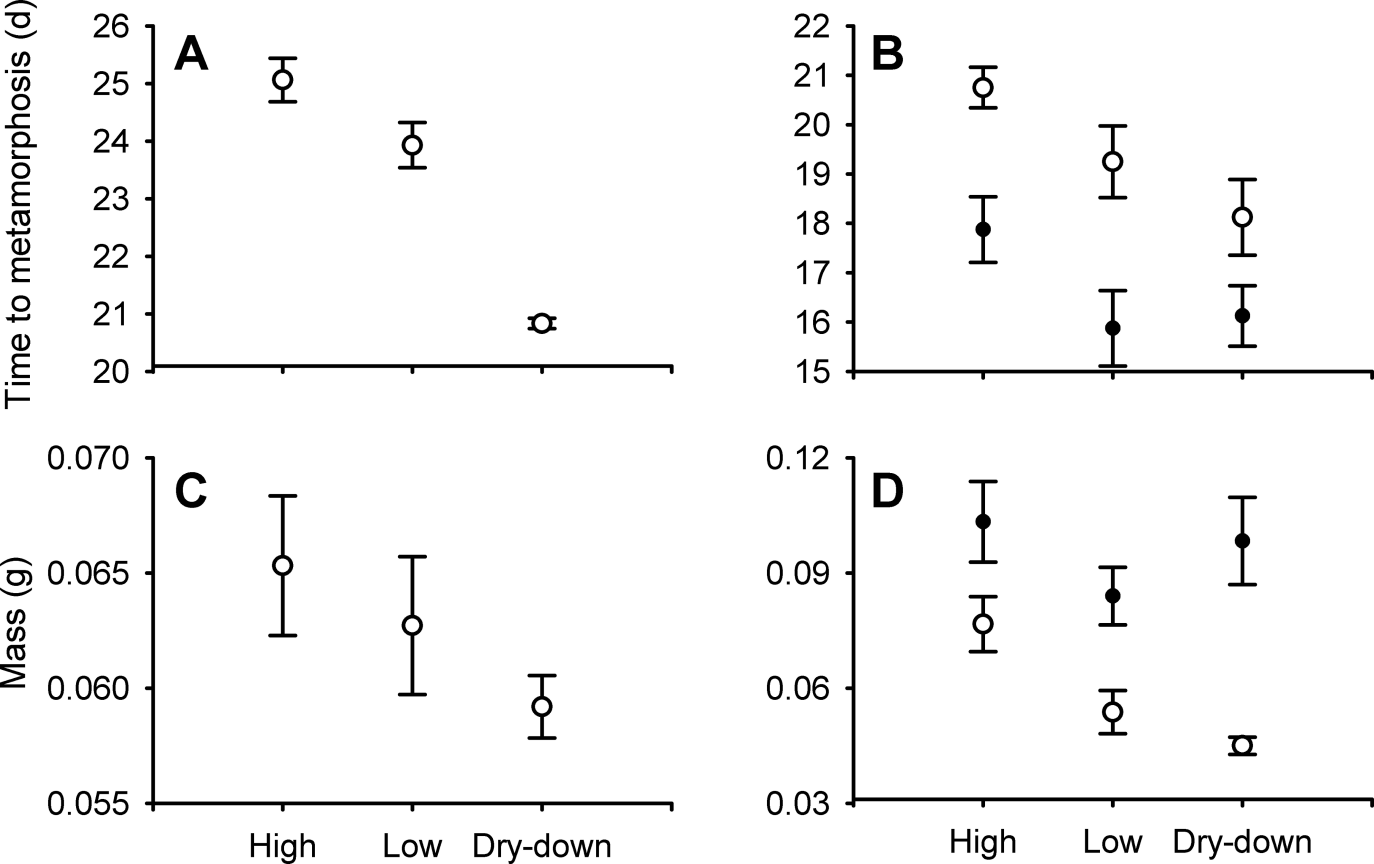
Effect of water depth and resource manipulations on time and size at metamorphosis. Effect of water depth and resource manipulations on mean time to metamorphosis and mean size at metamorphosis (± 1 SE) in the field mesocosm experiment (A, C) and laboratory experiment (B, D) in *Physalaemus pustulosus*. High depth were maintained in 10 cm of water (*n* = 9), low depth were maintained in 2 cm of water (*n* = 9), and dry-down in 10 cm to 1 cm of water (*n* = 10). Laboratory animals in high resource treatments (filled symbols) received twice as much resource (fish flakes) compared to animals in low resource treatments (open symbols).

**Figure 2:**
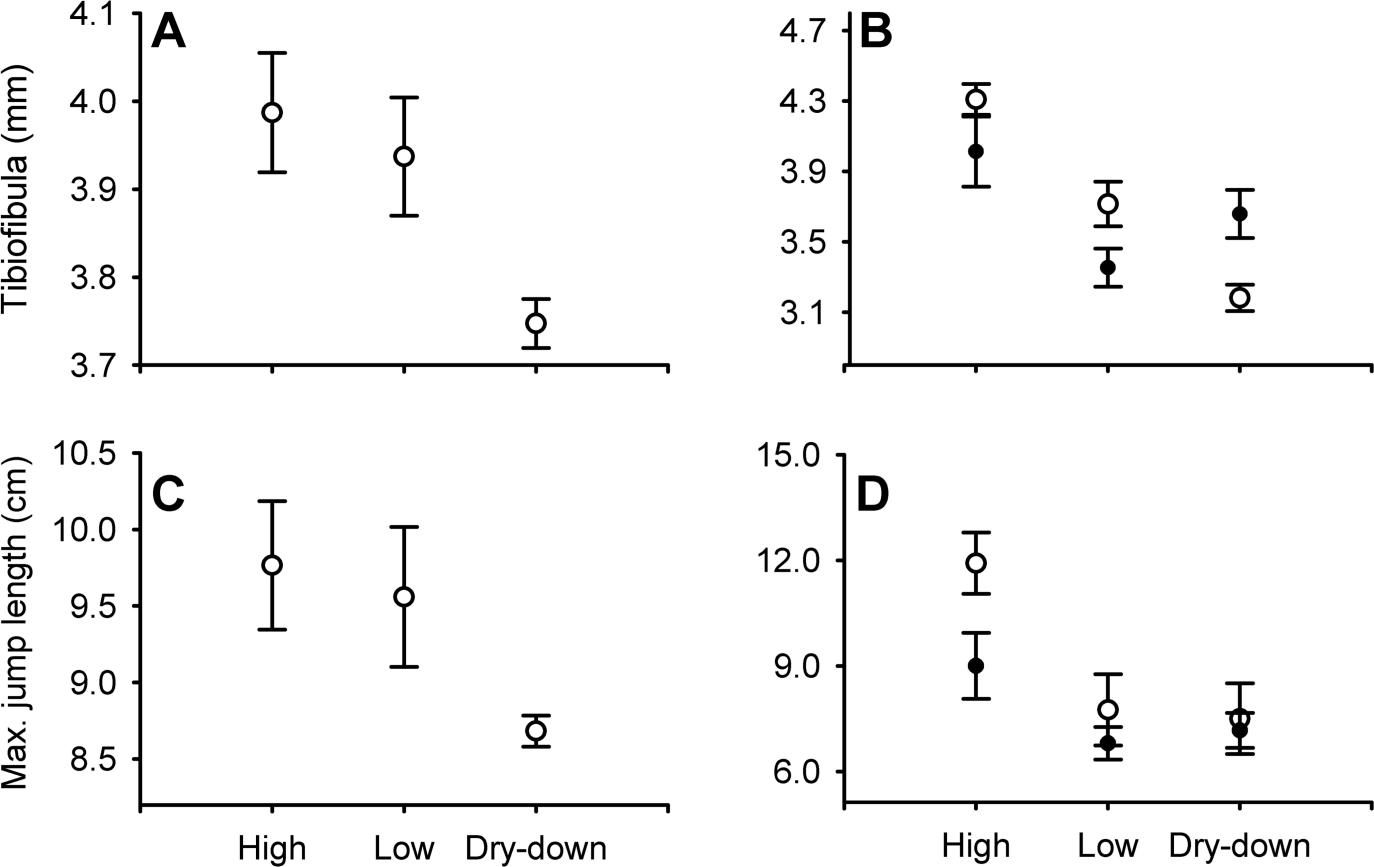
Effect of water depth and resources manipulations on tibiofibula length and jumping performance. Effect of water depth and resource manipulations on mean tibiofibula length and mean jumping performance (± SE) in the field mesocosm experiment (A, C) and laboratory experiment (B, D) in Túngara metamorphs. In (B) and (D), open symbols represent low resource treatments and filled symbols represent high resource treatments.

**Figure 3:**
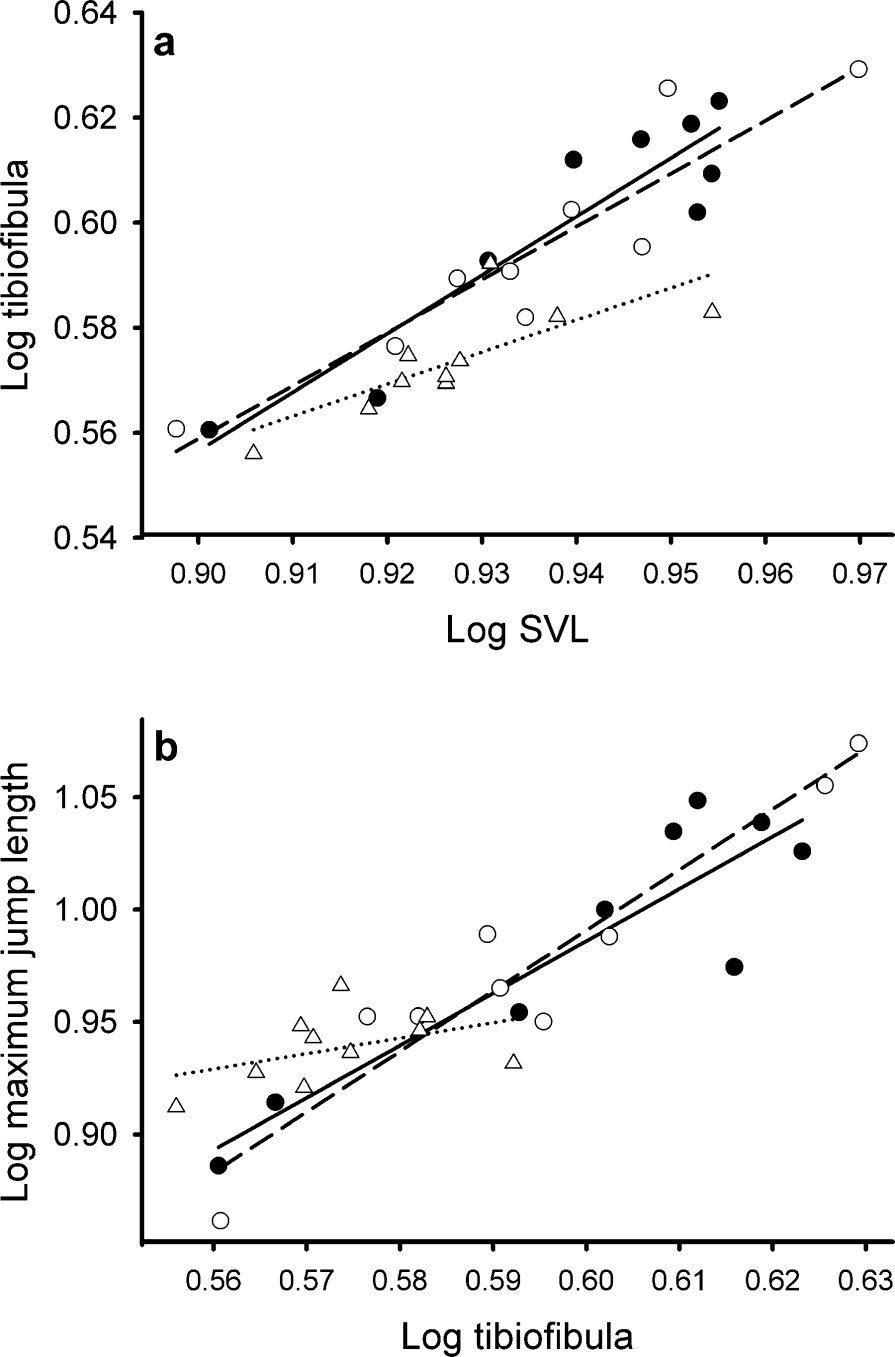
Relationship between morphology and jumping performance in the field mesocosm experiment. Relationship between (A) tibiofibula length and SVL and (B) maximum jump length and tibiofibula length as a function of water depth (high depth treatments (● solid line), low depth treatments (○, dotted line), and dry-down treatments (Δ, medium dashed line)) in the field mesocosm experiment.

Desiccation altered several aspects of metamorphic phenotype (MANOVA Pillai = 0.80, *F*_8,46_ = 3.85, *P* = 0.02). Desiccation affected time to metamorphosis (*F*_2,25_ = 7.90, *P* = 0.002), with frogs from dry-down treatments emerging 16% earlier (∼4 days) than constant high (*P* = 0.002) and 13% earlier (∼3 days) than constant low depth frogs (*P* = 0.03; Fig. 1A). Desiccation treatment did not affect metamorph size (SVL (mm): *F*_2,25_ = 1.25, *P* = 0.30; mass (g): *F*_2,25_ = 1.53, *P* = 0.24; Fig. 1C), but did affect absolute tibiofibula length (*F*_2,25_ = 5.30, *P* = 0.01, Fig. 2A). Specifically, the tibiofibula of dry-down frogs were 6% shorter than constant high depth treatments (*P* = 0.013) and marginally 4.8% shorter compared to constant low depth treatments (*P* = 0.057, Fig. 2A). Tibiofibula length increase with SVL and dry-down frogs had shorter size independent tibiofibula length (Table 2 and Fig. 3A). ANCOVA revealed that none of the interaction terms involving covariates were significant for maximum jump length (*P* > 0.06). Non-significant terms were removed from analyses and we recalculated the remaining effects (Table 2).

**Table 1:** MANOVA results for the laboratory experiment. Results of a MANOVA that examined the effect of water depth (low depth, high depth, and decreasing water depth) and resource manipulations (high food levels, low food levels) on metamorphic phenotype of Túngara metamorphs in the laboratory experiment. Statistically significant results are shown in bold.

Multivariate tests source	*df*	Wilks’ *F*	*P*
Resources	4,28	13.3	**<0.001**
Water depth	8,58	3.04	**<0.006**
Resources × water depth	8,58	1.29	<0.27

	Resources		Water depth		Resources × water depth	
Variable	*P*	*F*_1,31_	*P*	*F*_2,31_	*P*	*F*_2,31_
Larval duration	**<0.0001**	22.9	**0.0066**	39.0	0.99	0.015
SVL	**<0.0001**	31.9	**0.01**	2.85	0.17	1.85
Mass (Stage 46)	**<0.0001**	34.6	**0.03**	4.02	0.21	1.65
Tibiofibula	0.72	0.14	<0.001	17.6	**0.005**	6.21

**Table 2:** ANCOVA results for the field mesocosm experiment. Results from an ANCOVA to examine the effects of water depth (low depth, high depth and decreasing water depth) and SVL on the tibiofibula length of Túngara metamorphs in the field mesocosm experiment. Statistically significant results are shown in bold.

Factor	*df*	*F*	*P*
SVL	1	118.3	**<0.0001**
Water depth	2	6.92	**0.005**

Looking across the three treatments, there were no differences in maximum (*F*_2,25_ = 2.542, *P* = 0.098; Fig. 2C) or average jump distance (*F*_2,25_ = 2.483, *P* = 0.104). The best fitting stepwise linear regression model explained 83% of the variation in maximum jump distance (*F*_7,18_ = 18.47, *P* < 0.001) and retained terms for relative leg length (*F*_1,18_ = 19.46, *P* < 0.0003) and SVL (*F*_1,18_ = 103.92, *P* < 0.0001). In both the constant water depth treatments, increased tibiofibula length corresponded to higher maximum jump distance (high depth: *F*_1,8_ = 30.5, *P* < 0.001, *R*^2^ = 0.79, *m* = 0.26 SE = 0.047, *t*_7_ = 5.52, *P* < 0.001; low depth: *F*_1,7_ = 64.9, *P* < 0.001, *R*^2^ = 0.89, *m* = 0.29 SE = 0.037, *t*_7_ = 8.057, *P* < 0.001), while in dry-down treatments there was no relationship between tibiofibula length and maximum jump length (*F*_1,8_ = 1.80, *P* = 0.22, *R*^2^ = 0.08 SE = 0.058, *m* = 0.08, *t*_8_ = 1.34, *P* = 0.22, Fig. 3B). Results for average jump distance parallel those of maximum jump.

#### Laboratory experiment

We identified three extreme outliers out of 48 animals based on tibiofibula length as defined by Hoaglin & Welsch (1978) and Fox (1997) across three different treatments (low resource & high depth, high resource & low depth, high resource & dry-down) that had high leverage and influence (see Supplemental Information 3). These outliers were excluded from analysis. Analyses gave qualitatively similar patterns when outliers were not excluded. A 50% reduction in resources resulted in 17% (∼3 days) longer larval duration (*P* < 0.001; Fig. 1B, Table 1). Frogs from dry-down and low depth treatments emerged on average 10% earlier (∼2 days) than frogs from high depth treatments (*P* = 0.005, *P* = 0.018; Fig. 1B).

**Figure 4:**
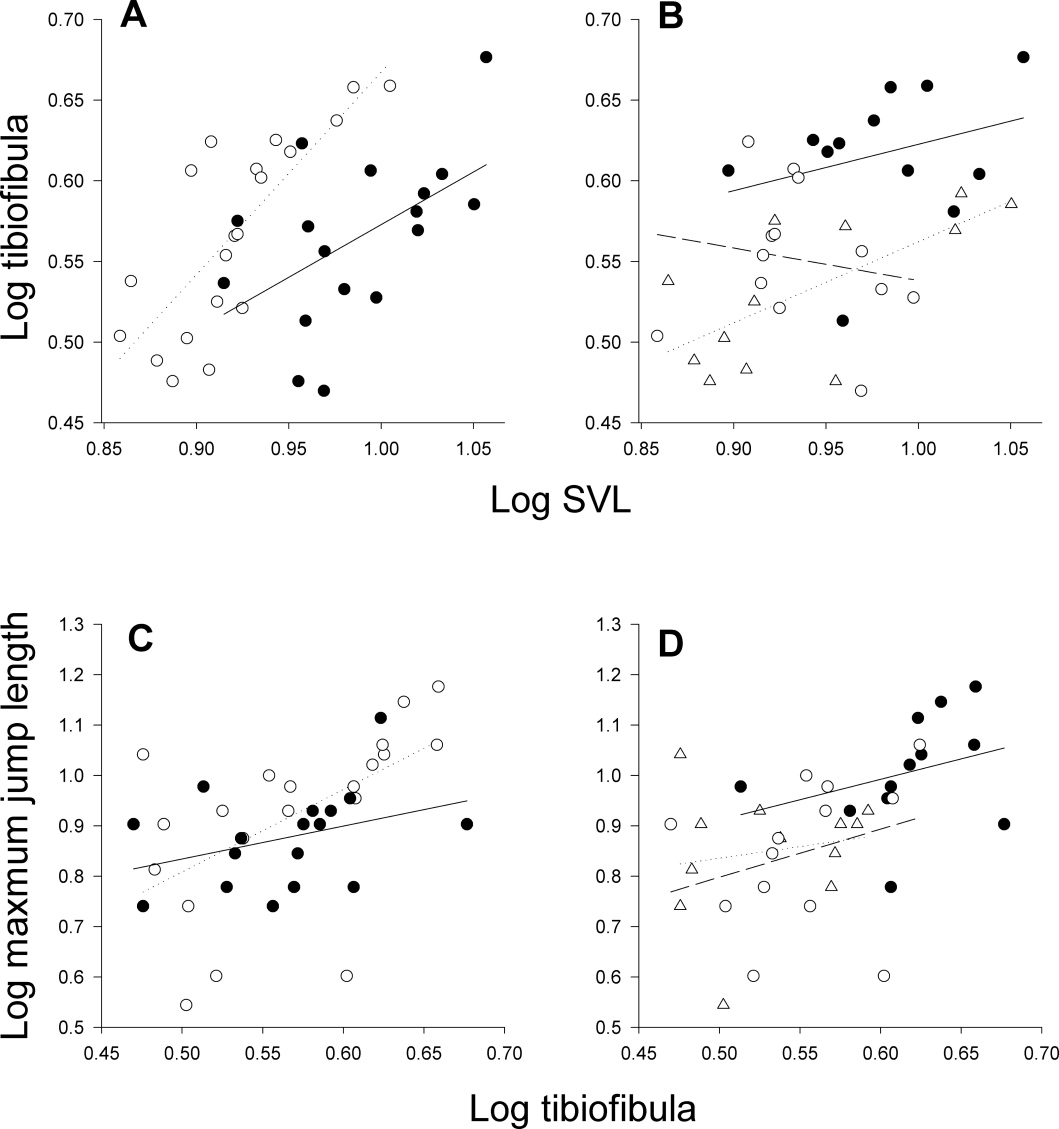
Relationship between morphology and jumping performance in the laboratory experiment. Relationship between tibiofibula length and SVL (A, B) and maximum jump length and tibiofibula length (C, D) as a function of resources levels (A, C) and water depth (B, D) in the laboratory experiment. In (A) and (C), low resources treatments are represented by open circles (dotted line), and high resource treatments are represented by a filled circles (solid line). In (B) and (D), high depth treatments are represented by filled circles (solid line), low depth treatments by open circles (dotted line), and dry-down treatments by open triangles (dashed line).

SVL and mass at metamorphosis were affected by both resources and water depth. Frogs from high resource treatments were 14% longer and 40% heavier than frogs from low resource treatments (*P* < 0.001, *P* < 0.001; Figs. 1D and 4A). Frogs from dry-down (*P* = 0.025) and low depth treatments (*P* = 0.02) had on average 10% smaller SVL than high depth frogs (Fig. 4B). Frogs from dry-down treatments had 20% smaller mass (g) than frogs from high depth treatments, although this effect was marginal (*P* = 0.07; Fig. 1D). Frogs from low depth treatments were 27% smaller in mass than frogs from high depth treatments (*P* = 0.03: Fig. 1D).

**Figure 5:**
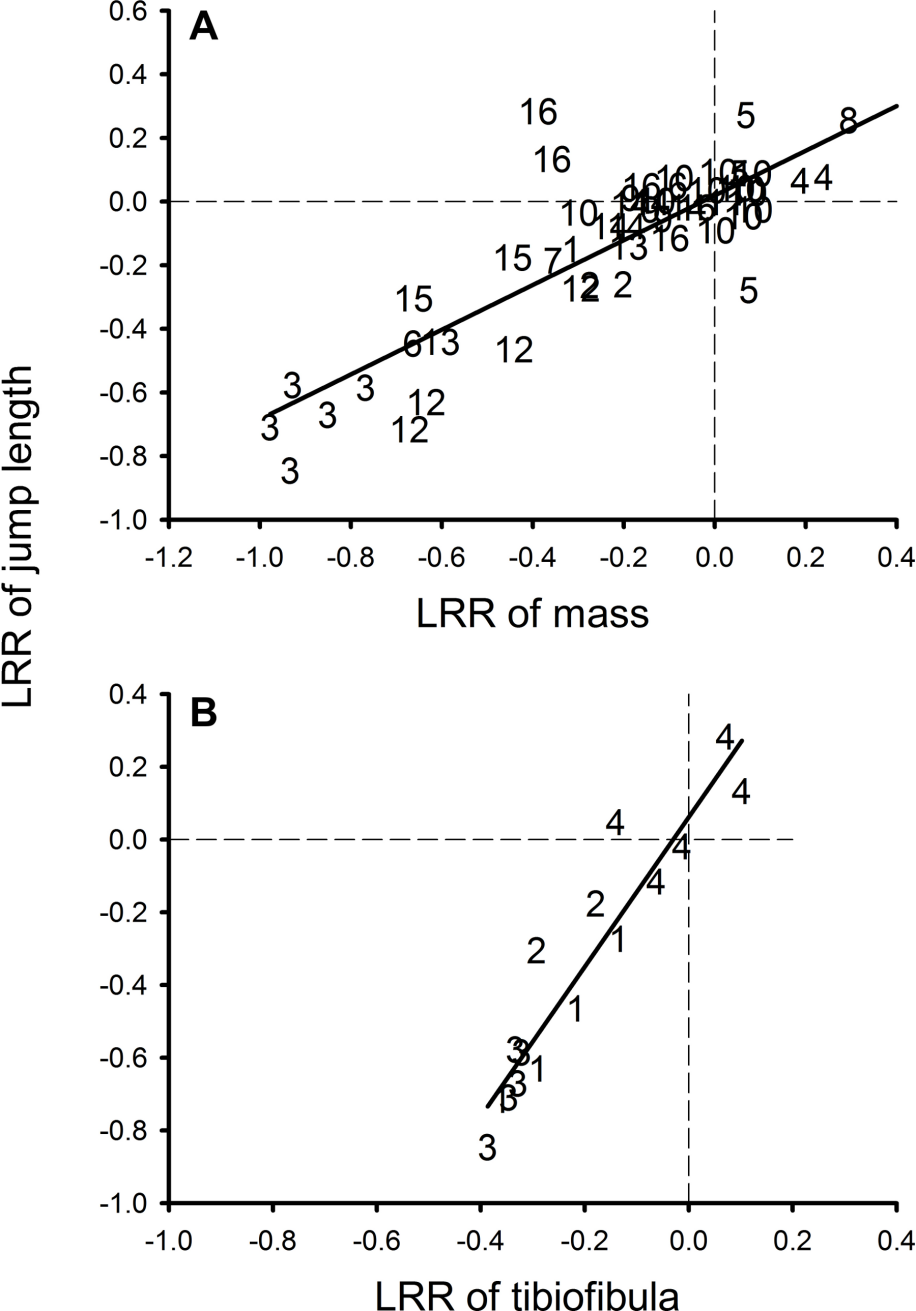
Relationship between log response ratio of mass and log response ratio of jumping performance in the meta-analysis. Relationship between log response ratio of mass and log response ratio of maximum jumping performance across 10 species (*m* = 0.65 SE = 0.07) (1) John-Alder & Morin (1990), (2) Goater, Semlitsch & Bernasconi (1993), (3) Tejedo, Semlitsch & Hotz (2000b), (4) Álvarez & Nicieza (2002), (5) Niehaus, Wilson & Franklin (2006), (6) Gomez-Mestre et al. (2010), (7)Johansson, Lederer & Lind (2010), (8) Hector, Bishop & Nakagawa (2012), (9) Todd et al. (2012), (10) Dahl et al. (2012), (11) Gibbons & George (2013), (12) Enriquez-Urzelai et al. (2013), (13) Cabrera-Guzmán et al. (2013), (14) Fan, Lin & Wei (2014), (15) J Charbonnier, 2010, unpublished data, (16) this study (A) and relationship between log response ratio of tibiofibula and log response ratio of maximum jumping performance across 7 species (*m* = 1.98 SE = 0.74) (1) Tejedo, Semlitsch & Hotz (2000b), (2) Enriquez-Urzelai et al. (2013), (3) J Charbonnier, 2010, unpublished data, (4) this study (B).

Absolute tibiofibula length was shaped by the interaction of resources and water depth (*P* = 0.005, Table 1 and Fig. 2B). In low resources treatments, frogs from dry-down treatments had 36% and 14% shorter tibiofibulas than high and low depth treatments respectively (*P* < 0.001, *P* = 0.04) but there was no such pattern in high resources treatments (Fig. 2B). In high resources, there was no difference in absolute tibiofibula length between dry-down frogs and either control treatment although low water depth frogs had 16% shorter tibiofibulas than high depth frogs (*P* = 0.023, Fig. 2B). Tibiofibula length increased with SVL and dry-down frogs had shorter size independent tibiofibula length than the constant high treatments across both resource treatments (Table 3 and Fig. 4B). There were no other interactive effects of resource manipulation and water depth on the other variables (Table 1).

**Table 3:** ANCOVA results for the laboratory experiment. Results from ANCOVA to examine the effects of water depth (low depth, high depth and decreasing water depth), resource levels (high and low) and SVL on the tibiofibula length of Túngara metamorphs in the laboratory experiment. Statistically significant results are shown in bold.

Factor	df	F	*P*
Water depth	2	18.8	**<0.0001**
Resources	1	0.26	0.61
SVL	1	14.2	**0.0009**
Water depth: resources	2	3.84	**0.035**
Water depth: SVL	2	0.44	0.65
Resources: SVL	1	0.003	0.95
Water depth: resources: SVL	2	1.332	0.28

In the laboratory experiment, decreasing and constant low water depth negatively affected maximum jumping performance (*F*_2,31_ = 8.53, *P* = 0.001; Fig. 2D). Frogs from dry-down treatments had 33% shorter maximum jump distance (*P* = 0.004) and frogs from low depth treatments had 23% shorter maximum jump length than frogs from constant high depth treatment (*P* = 0.002, Fig. 2D). Results for average jump distance parallel those of maximum jump. The best fitting stepwise linear regression model explained 34% of the variation in maximum jump distance (*F*_5,31_ = 3.227, *P* = 0.02) and only relative tibiofibula length was significant (*F*_1,31_ = 11.0749, *P* = 0.002). Main effects and two-way interactions were retained within the model but were not significant (all *P* > 0.06). As in the field experiment, in the constant treatments there tended to be a stronger relationship between tibiofibula and jumping distance than in the dry-down treatment (Fig. 4D); however, tibiofibula was not a significant predictor of jump distance within any of the desiccation treatments.

#### Meta-analysis—carryover effects on juvenile performance

Our literature search yielded 23 studies from 1990 to 2013 across 18 different species (Supplemental Information 1). Papers which used the same data were only included once. These studies manipulated various aspects of the larval environment and then measured jumping performance immediately after metamorphosis or shortly thereafter (Gosner Stage 46 (0 days) days—138 days, Supplemental Information 1). Results did not differ between studies that measured performance immediately after metamorphosis (0 days) or shortly thereafter. Papers contributed multiple effect sizes from manipulating different factors and using multiple species. We were unable to extract effect sizes from some studies if the data were not available (see Supplemental Information 1). If the juvenile environment was manipulated (e.g., Goater, Semlitsch & Bernasconi, 1993), only control treatments were included.

From these published studies, we extracted 46 effect size estimates on mass and jump length. We also included one unpublished data set and our present study in the analyses for a total of 53 effect sizes from 12 studies and 1 unpublished data sets across 10 species. Buckley, Michael & Irschick (2005) was a statistical outlier and was excluded from further analyses. The focal species in Buckley, Michael & Irschick (2005), the coquí frog, is a direct developer and early-hatched individual retain a tail which may impede jumping performance (Capellán & Nicieza, 2007). We first conducted analyses on all datasets, as all studies reported mass and maximum jump length. We then ran parallel analyses without the data from this study (presented above) to ensure our study was not driving results of the meta-analysis. Due to our small sample size, we limited our analyses to the relationships between mass, limb length and jumping performance.

Across all studies, LRR mass at metamorphosis explains 71% of the variation in LRR of jump length (*F*_1,50_ = 131.6, *P* < 0.0001, coefficient = 0.70, SE = 0.06, Fig. 5A). When our study was removed, the best fitting linear regression model explained 82% of the variation (*F*_1,45_ = 229.07, *P* < 0.0001, coefficient = 0.72, SE = 0.05) but the slope of the main effect (mass) was similar (0.70 versus 0.73).

Only a subset of data reported changes in morphology, and different studies measured different morphological characteristics of the hindlimb (e.g., femur, tibiofibula; see Supplemental Information 1). Since our objective was to place the results of our empirical data within the context of other studies and because most extractable hindlimb data were for tibiofibula length, we focused on the relationship between tibiofibula length, mass and jumping performance. We chose to include mass (rather than SVL) so that these analyses could be compared with our first analysis comparing mass and maximum jump length. We analyzed data from 5 species from 2 published data sets and one unpublished data set (for 16 effect sizes, plus 12 from this study, totaling 28 effect sizes). The best fitting linear model explained 86% of the variation in LRR jump length (*F*_3,24_ = 56.02, *P* < 0.0001, Fig. 5B) and retained terms for LRR tibiofibula (*F*_1,24_ = 63.24, *P* < 0.001, coefficient = 1.45, SE = 0.36), and a LRR mass by LRR tibiofibula interaction (*F*_1,24_ = 5.40, *P* = 0.03). When we excluded data from our study, we found that only tibiofibula impacted jumping performance (*F*_3,12_ = 37.73, *P* < 0.0001, *R*^2^ = 0.88) and retained only the LRR tibiofibula term (*F*_1,12_ = 6.649, *P* = 0.02, coefficient = 1.98, SE = 0.74).

## Discussion

Our objective was to manipulate the larval environment to induce life history switchpoint plasticity and quantify the consequences on size, morphology and jumping performance. Across outdoor mesocosm and laboratory experiments, both low and decreasing water levels (dry-down) hastened the metamorphic switch point. These results are consistent with previous empirical studies that demonstrate both low water levels (Denver, Mirhadi & Phillips, 1998; Spieler, 2000; Székely, Cogălniceanu & Tudor, 2010; Fan, Lin & Wei, 2014) and drying environments shorten larval duration (Newman, 1992; Richter-Boix, Llorente & Montori, 2006; Richter-Boix, Tejedo & Rezende, 2011; Michimae & Emura, 2012; Gomez-Mestre, Kulkarni & Buchholz, 2013).

Larval duration was also influenced by resource availability. In the laboratory experiment, resource-limited larvae had lower growth rates and required more time to complete metamorphosis across water level manipulation treatments. This result is consistent with the Wilbur–Collins (1973) model of metamorphosis which predicts that larvae in resource limited environments take longer to reach a threshold minimum body size (Enriquez-Urzelai et al., 2013).

Gomez-Mestre et al. (2010) predicts that environmental characteristics which primarily influence growth rates (e.g., resource availability) will have a greater impact on size at metamorphosis than environment characteristics that influence differentiation (e.g., pond drying). Our results support this hypothesis as resource availability was the biggest determinant of size at metamorphosis in our laboratory experiment. Metamorphic size was further reduced in dry-down and low water depth treatments across resource manipulation treatments, but this effect was smaller than the impact of resource manipulation. In previous experiments that manipulate both resource levels and pond-drying, resource limited animals fail to accelerate development in response to drying ponds (Newman, 1989; Loman, 2002; Enriquez-Urzelai et al., 2013). We do not think this is the case in our experiments, as larvae successfully completed metamorphosis and survival was high. Our results support the theory that resource availability (when sufficient to sustain growth) is the largest determinant of body size. We cannot test the influence of resource availability in our field mesocosm experiment, as we only manipulated water levels. We did not find that pond-drying influenced metamorphic size in frogs from our field study. When compared to laboratory animals, animals from our field study had lower growth rate and emerged smaller. We postulate that overall low growth rates impeded any significant effects of pond-drying on body size in the field mesocosm experiment.

The larval environment also induced changes in the absolute and relative leg length. In the field experiment, frogs from dry-down treatments had both absolute and proportionally shorter legs. This is consistent with empirical and theoretical work that suggest that accelerated development in ephemeral environments reduce tibiofibula length (Morey & Reznick, 2004; Richter-Boix, Llorente & Albert, 2006; Tejedo et al., 2010; Gomez-Mestre, Kulkarni & Buchholz, 2013). Like body size, the relative length of the limbs is a summation of past growth and differentiation processes (Blouin, 1991). In the laboratory experiment, absolute and relative leg length were dependent on both resource availability and water manipulation. On average, frogs in low resource treatments had longer absolute and relative leg lengths than frogs from high resource treatments, both because they had longer absolute legs and because of their shorter body length. Across resource manipulation treatments, frogs in low depth and dry-down treatments had shorter absolute and relative leg length, consistent with the results from our field study.

We were interested in how this variation in size and morphology would translate to differences in juvenile jumping performance. We predicted that small juveniles with short relative leg length would have the poorest jumping performance. In the field mesocosm experiment, juveniles emerging from drying ponds had higher size independent performance despite their shorter absolute jump length. In other words, they jumped farther than similarly sized frogs from other treatments that had proportionally longer legs.

In the laboratory experiment, the resource manipulation generated much greater size variation in length and mass across treatments than observed in the field experiment, allowing us to explore the relationship between morphology and performance across a larger range of sizes. Although there were large differences in mass and SVL between treatments these differences in mass did not translate to parallel patterns in maximum jump length. Juveniles from low resource treatments (lighter and shorter in length) had similar jumping performance to heavier and longer juveniles from high resource treatments, potentially because of their proportionally longer legs. Low resources unexpectedly resulted in smaller but better performing juveniles. Although we do not suggest these differences are adaptive, they do suggest that reductions in metamorph mass may not always lead to decreases in jumping performance. Larvae in resource limited, ephemeral environments had comparable performance to animals in resource rich environments, despite differences in size and morphology.

To place these results into a broader context, we quantitatively summarized past studies that manipulated the larval environment in anurans and reported effects on size, morphology and jumping performance. These studies manipulated a range larval or larval environment characteristics including hatching time, larval density, resources, and temperature (see Supplemental Information). Across studies, increases in juvenile mass were correlated with increases in jump length. Larval environment manipulations that generated large size differences in mass resulted in larger changes in performance. Manipulations in most experiments to date have resulted in both lower mass and lower jumping performance. This synthesis focuses on laboratory and mesocosms experiments but similar patterns have been observed in field enclosure studies (see Ficetola & De Bernardi, 2006; Boes & Benard, 2013). This analysis includes studies that monitored jumping up to three months after metamorphosis, suggesting that in the absence of compensatory growth, differences in mass and jumping performance may persist, at least in the short term. In anurans, larger mass at metamorphosis is likely positively linked to fitness (Smith, 1987; Berven, 1990; Scott, 1994) and enhanced jumping performance may be one of the mechanisms resulting in higher survival of larger animals (Cabrera-Guzmán et al., 2013).

Our subsequent analysis incorporated how limb morphology may interact with body size to shape performance. Across five species, reduction in absolute tibiofibula length resulted in large decreases in jumping performance. The relationship between absolute limb length and jumping performance had a steeper slope than that of mass and jumping, suggesting that, for an equivalent proportional change, tibiofibula has a greater impact on performance than mass. If possible, both mass and hindlimb length should be measured when relating past environmental variation to jumping performance. While mass at metamorphosis provides a robust estimator of jumping performance, taking into account the size of the limbs may more accurately capture the effects of past histories on performance. For instance, in our laboratory experiment, knowing the length of the hindlimb helped explain the comparable performance of low resource frogs. Longer hindlimbs may buffer against the effects of reduced size at metamorphosis, resulting in similar performance. For juvenile animals, locomotive advantages may be particularly important as they face the same predators as adults, but are smaller, naïve and still developing (Carrier, 1996). We suggest that the relationship between limb morphology and jumping performance in juvenile anurans is flexible and can buffer against the negative effects of small size on locomotor performance.

These results of our meta-analysis combined with our empirical data on the Túngara frog showcases that the larval environment can mediate important aspects of juvenile performance by inducing changes in both mass and morphology. Across both experiments, plasticity in the timing of metamorphosis resulted in significant phenotypic effects on the performance of juvenile frogs. We conclude that variation in larval environments alter the relationships between body size, limb length, and locomotor performance. Our synthesis of current available data supports that the relationship between body size, limb length and jumping performance is flexible and influenced by variation in the larval environment. Together, these data reinforce that life stages are interconnected through phenotypic traits which manifest themselves across the life history switch point.

## Supplemental Information

10.7717/peerj.1268/supp-1 Supplemental Information Effect of water and resource manipulations on larval growth Effect of water depth and resource manipulations on mean larval growth during the first two weeks (±1 SE) in the field mesocosm experiment (A) and laboratory experiment (B) in *Physalaemus pustulosus*.

10.7717/peerj.1268/supp-2 Supplemental Information 1 Table listing studies and contributing effect sizes to the meta-analysis Studies which manipulated the larval environment and report changes in mass, tibiofibula length, and jumping performance. Y, Year; E.S.M., Effect size of mass; E.S.T.F., Effect size of tibiofibula size; HL, Hindlimb length; FL, Femur length; TF, Tibiofibula lengh; FOL, Foot length; Stage; Stage at which performance was measured; GS, Gosner Stage; UE, Until exhausted; *, inferred from text; N, jump, number of jump.

10.7717/peerj.1268/supp-3 Supplemental Information 2 PRISMA Flow Diagram

10.7717/peerj.1268/supp-4 Supplemental Information 3 Supplemental version of Figure 4 including the three outlier data points

10.7717/peerj.1268/supp-5 Supplemental Information 4 PRISMA 2009 Checklist

## Additional Information and Declarations

### Field Study Permissions

The following information was supplied relating to field study approvals (i.e., approving body and any reference numbers): Permiso No. SE/A-13-11 Autoridad Nacional del Ambiente de Panamá.

### Data Availability

The following information was supplied regarding data availability: Dryad: 10.5061/dryad.9855v.
