# Supplementary figures and images for: Consequences of life history switch point plasticity for juvenile morphology and locomotion in the Túngara frog
Source: PeerJ. 2015 Sep 22;3:e1268. doi: 10.7717/peerj.1268 (PMC4582954; doi:10.7717/peerj.1268)

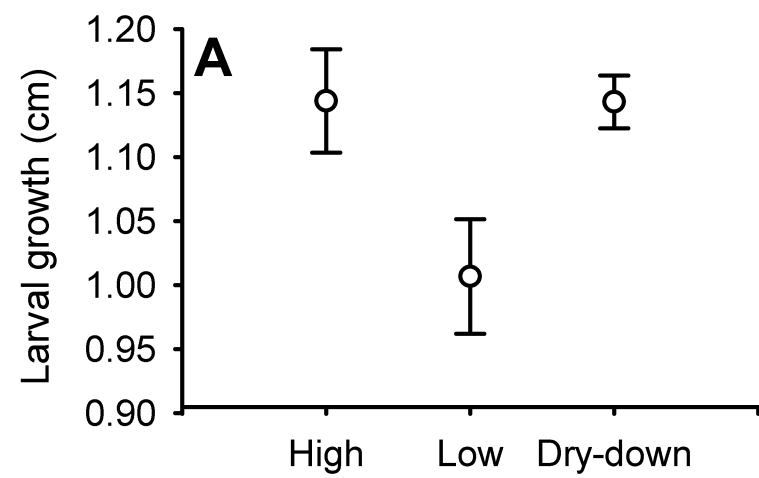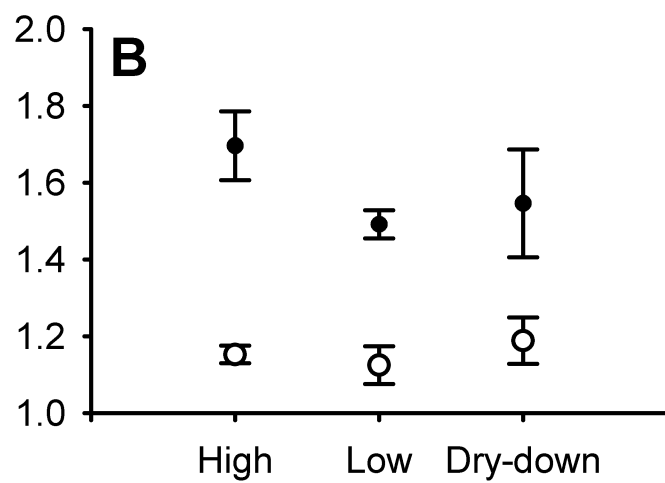

Supplement: Supplemental Information — Effect of water depth and resource manipulations on mean larval growth during the first two weeks (±1 SE) in the field mesocosm experiment (A) and laboratory experiment (B) in Physalaemus pustulosus. [file peerj-03-1268-s001.pdf]

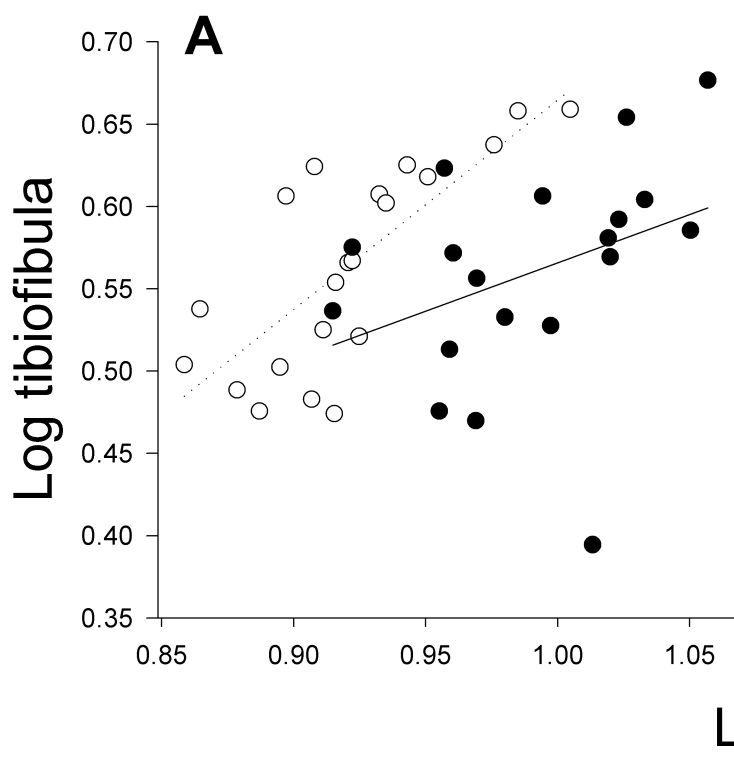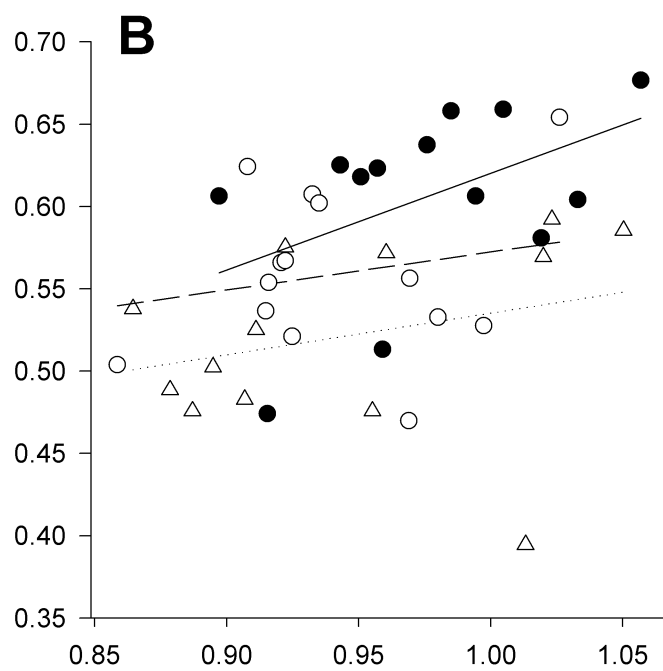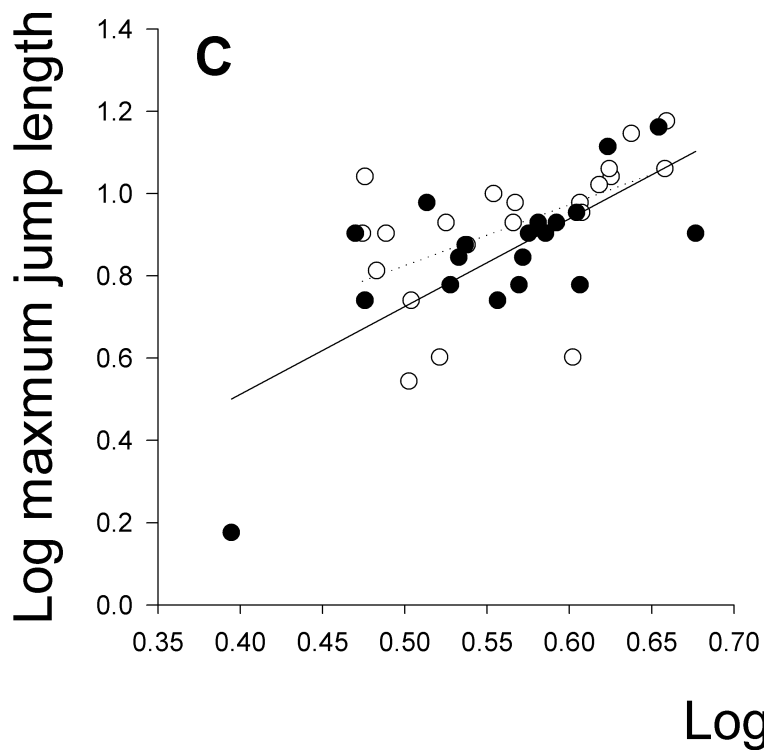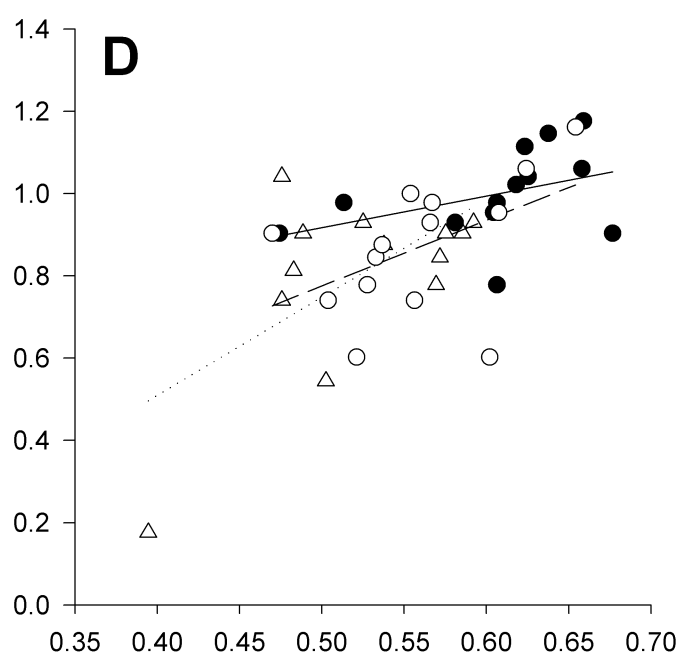

Supplement: Supplemental Information 3 [file peerj-03-1268-s004.pdf]
