# Supplementary material for: Consequences of life history switch point plasticity for juvenile morphology and locomotion in the Túngara frog
Source: PeerJ. 2015 Sep 22;3:e1268. doi: 10.7717/peerj.1268 (PMC4582954; doi:10.7717/peerj.1268)
Supplement: Supplemental Information 1 — Studies which manipulated the larval environment and report changes in mass, tibiofibula length, and jumping performance. Y, Year; E.S.M., Effect size of mass; E.S.T.F., Effect size of tibiofibula size; HL, Hindlimb length; FL, Femur length; TF, Tibiofibula lengh; FOL, Foot length; Stage; Stage at which performance was measured; GS, Gosner Stage; UE, Until exhausted; *, inferred from text; N, jump, number of jump. [file peerj-03-1268-s002.docx]

| **Authors** | **Y** | **Species** | **Venue** | **Treatment** | **# E.S**  **M** | **# E.S.**  **TF** | **HL** | **FL** | **TF** | **FOL** | **Stage** | **N. jump** |
| --- | --- | --- | --- | --- | --- | --- | --- | --- | --- | --- | --- | --- |
| John-Alder &Morin | 1990 | *B woodhousii fowleri* | Field | Density | 1 | 0 | N | N | N | N | G.S. 45 +2d | 4-8 |
| Goater et al. | 1993 | *B. bufo* | Field | Density | 2 | 0 | N | N | N | N | 14, 28d | 4 |
| Semlitsch et al. | 1999 | *R. spenocephala* | Field | Density | 0 | 0 | N | N | N | N | 25-40d | 3-5 |
| Semlitsch et al. | 1999 | *R. blairi* | Field | Density | 0 | 0 | N | N | N | N | 25- 40d | 3-5 |
| Tejedo et al. | 2000 | *R. esculenta* | Field | Density | 1 | 1 | Y | Y | Y | Y | 2-3d | ≥ 5 |
| Tejedo et al. | 2000 | *R. lessonae* | Field | Density | 3 | 3 | Y | Y | Y | Y | 2-3d | ≥ 5 |
| Tejedo et al. | 2000 | *R. ridibunda* | Field | Density | 1 | 1 | Y | Y | Y | Y | 2-3d | ≥ 5 |
| Van Buskirk & Saxer | 2001 | *R. ridibunda* | Field | Predator Presence | 0 | 0 | N | Y | Y | N | G.S. 45 | NA-UE |
| Alverez & Nicieza | 2002 | *D. galganoi* | Lab | Diet quality  + Temp | 3 | 0 | N | N | N | N | 7-10d | 5 |
| Buckley et al. | 2005 | *E. coquí* | Lab | Hatching time | 1 | 0 | Y | N | N | N | G.S. 15+1hr | 3-20 |
| Ritcher - Boix et al. | 2006 | *P. punctatus* | Lab | Drydown | 0 | 0 | N | Y | Y | Y | G.S.46 +1d | NA-UE |
| Niehaus et al. | 2006 | *L. peronii* | Lab | Temp. | 3 | 0 | Y | N | N | N | G.S. 42*,63, 133d | ≥ 3 |
| Nicieza et al. | 2006 | *D. galganoi* | Lab | Diet quality + Predator Cue | 0 | 0 | N | Y | Y | N | 7-10d | 5 |
| Capellan & Nicieza | 2007 | *R. temporaria* | Lab | Egg predator | 0 | 0 | N | N | Y | N | 14,21,28d | 5 |
| Capellan & Nicieza | 2007 | *R.temporaria* | Lab | Larval Predator | 0 | 0 | N | N | Y | N | 7 d | 5 |
| Orizaola & Laurila | 2009 | *R. lessonae* | Lab | Temp. | 0 | 0 | N | N | Y | N | G.S. 46 | NA |
| Gomez-Mestre et al. | 2010 | *A. callydrias* | Lab | Temp +Food | 2 | 0 | Y | N | N | N | G.S 46* | NA-UE |
| Johansson et al. | 2010 | *R. temporaria* | Lab | Water level | 1 | 0 | N | Y | Y | Y | G.S 46 | 3 |
| Hector et al. | 2011 | *L. ewingii* | Lab | Food | 1 | 0 | N | N | N | N | 97,131,138d | NA |
| Todd et al. | 2011 | *B. americanus* | Lab | Mercury exposure | 3 | 0 | N | N | N | N | G.S 46* | 4 (mean) |
| Dahl et al. | 2012 | *R. temporaria* | Lab | Food & Predator Cue | 12 | 0 | N | N | N | N | G.S 46 | NA |
| Gibbons & George | 2013 | *A. callydrias* | Lab | Hatching time | 1 | 0 | N | N | N | N | G.S 45 | NA-UE |
| Enriquez-Urzelai et al. | 2013 | *D. pictus* | Lab | Water level + Food | 4 | 4 | Y | Y | Y | Y | G.S 46* | 5 |
| Cabrera-Guzman et al. | 2013 | *R. marina* | Field | Density | 2 | 0 | N | N | N | N | G.S.45* | 3 |
| Fan et al. | 2013 | *H. chinensis* | Field | Water level | 5 | 0 | Y | N | N | N | G.S 45* | ≥ 10 |
