# Supplementary material for: Consequences of life history switch point plasticity for juvenile morphology and locomotion in the Túngara frog
Source: PeerJ. 2015 Sep 22;3:e1268. doi: 10.7717/peerj.1268 (PMC4582954; doi:10.7717/peerj.1268)
Supplement: Supplemental Information 2 [file peerj-03-1268-s003.pdf]

PRISMA 2009 Flow Diagram

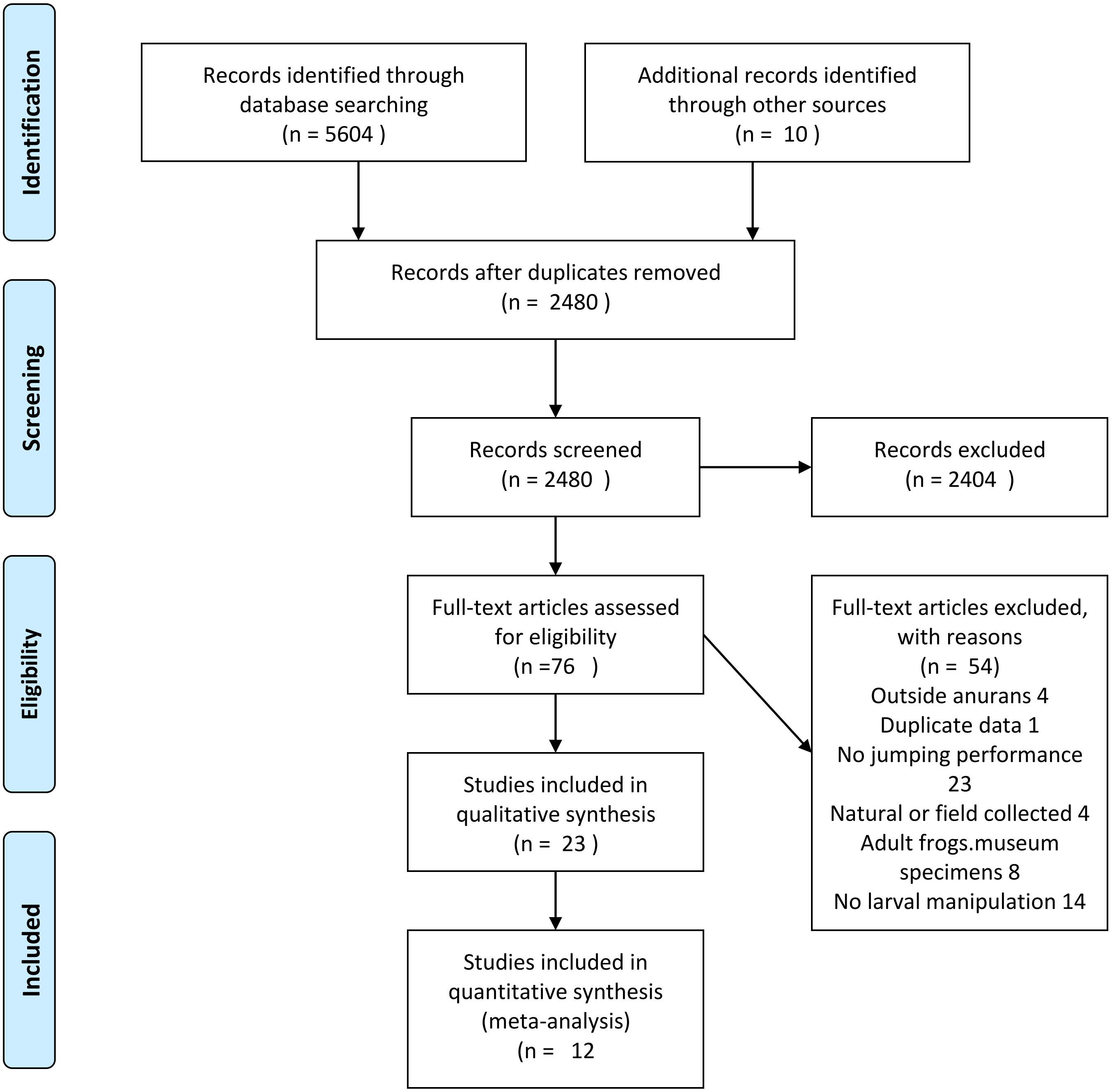

From: Moher D, Liberati A, Tetzlaff J, Altman DG, The PRISMA Group (2009). *P*referred *R*eporting *I*tems for *S*ystematic Reviews and *M*eta-*A*nalyses: The PRISMA Statement. PLoS Med 6(6): e1000097. doi:10.1371/journal.pmed1000097

For more information, visit www.prisma-statement.org.
